# Global Research Trends and Hotspots on Mitochondria in Acute Lung Injury from 2012–2021: A Bibliometric Analysis

**DOI:** 10.3390/ijerph20010585

**Published:** 2022-12-29

**Authors:** Song Hu, Wenyu Zhou, Sheng Wang, Zhuoran Xiao, Quanfu Li, Huanping Zhou, Meiyun Liu, Huimin Deng, Juan Wei, Wanli Zhu, Hao Yang, Xin Lv

**Affiliations:** Department of Anesthesiology, Shanghai Pulmonary Hospital, School of Medicine, Tongji University, Shanghai 200433, China

**Keywords:** mitochondria, acute lung injury, acute respiratory distress syndrome, bibliometric analysis, CiteSpace, VOSviewer

## Abstract

Background: Acute lung injury (ALI)/acute respiratory distress syndrome (ARDS) is a clinical syndrome associated with mitochondria and lacks effective preventive and therapeutic measures. This bibliometric study aims to gain insight into the scientific findings regarding mitochondria in ALI/ARDS. Methods: We retrieved the Science Citation Index Expanded (SCIE) of the Web of Science Core Collection (WoSCC) for mitochondria in ALI/ARDS publications from 2012–2021. VOSviewer, CiteSpace (5.8. R3) and Bibliometrix (3.1.4) R package were used for further analysis and visualization. Result: A total of 756 English-language articles and reviews were identified. The annual number of publications presented a rapidly developing trend. China was the most productive and cited country, and the USA had the greatest impact. In the keyword co-occurring network, the terms “acute lung injury”, “oxidative stress”, “inflammation”, “mitochondria” and “apoptosis” occurred most frequently. The co-citation network revealed that #1 mesenchymal stromal cell and #3 endothelial cell had the most bursts of citations. In addition, research hotspots have shifted from “potential therapeutic treatments” and “mitochondrial DNA (mtDNA)” to “endothelial cell” and “mesenchymal stromal cell (MSC)”. Conclusion: This bibliometric analysis reveals the research directions and frontier hotspots of mitochondria in ALI/ARDS, which has shown a rapid growth trend in annual publication numbers. mtDNA, mitophagy, and apoptosis have been the most active research areas, while studies on mitochondrial transfer in stem cells have become a hot topic in recent years.

## 1. Introduction

Acute lung injury (ALI) is a clinical syndrome caused by a variety of direct or indirect pathogenic factors and accompanied by extensive lung inflammation, which is characterized by acute diffuse alveolar injury, congestion, increased alveolar capillary permeability, inflammatory cell infiltration and an impaired blood–gas barrier. The clinical manifestation is hypoxic respiratory failure, which can further develop into acute respiratory distress syndrome (ARDS), and 80% of ALI/ARDS cases are caused by severe sepsis [1,2]. According to a research report, there are approximately three million cases of ARDS each year worldwide, accounting for 10% of intensive care unit (ICU) patients and a 35–40% mortality rate, which has become a global public health problem that seriously endangers human health [3,4]. In clinical practice, the main focus is on symptomatic supportive treatment, and there are no effective measures to prevent or treat septic lung injury [5,6]. There is a current necessity to further understand the pathophysiological processes and mechanisms of ALI/ARDS and to identify new therapeutic approaches to improve the survival and prognosis of ALI/ARDS patients [7]. As an important organelle for cellular biosynthesis, bioenergy and signaling, mitochondria play an important role in cellular adaptation to internal environmental homeostasis and maintenance of normal physiological functions [8,9,10,11,12,13]. Various endogenous and exogenous stresses affect crucial processes of mitochondrial homeostasis, such as the mitochondrial redox system, oxidative phosphorylation, mitochondrial biogenesis and mitophagy, which disrupt mitochondrial function [14,15]. Recent studies have shown that multiple aspects of mitochondrial biology are key determinants in the development and progression of ALI/ARDS, including mitochondrial dynamics and mitophagy, which ensure physiological responses to stresses by removing excess mitochondrial ROS (mtROS), mtDNA and other relevant factors [16,17]. In addition, mitochondria are highly susceptible target organelles in the early stages of sepsis, and their damage and dysfunction are closely related to the prognosis of septic lung injury. It is thus clear that mitochondria are critical cellular components in the developmental process of ALI/ARDS. Therefore, deciphering the mitochondria-related molecular mechanisms and modulatory targets in ALI/ARDS progression will contribute to our inspiration for potential new therapies.

Bibliometrics is a field of study in library and information science. As a new method of statistical analysis, it plays an important role in analyzing impacts and trends [18]. Therefore, bibliometrics is gradually gaining widespread attention and recognition [19]. By analyzing the characteristics of databases and the literature itself, bibliometrics can predict the trends of a field and reveal key research directions. In addition, it can be used as an auxiliary guidance strategy for scientific research and can play a decision-making role in research development [20]. In recent years, bibliometric analysis has been increasingly applied to clinical medicine, preventive medicine and alternative medicine [21,22,23,24]. However, no bibliometric studies have been conducted on mitochondria in ALI/ARDS. Understanding the current status, focus areas, and future prospects of mitochondria in ALI/ARDS will help explore the intrinsic relationship between the discipline’s knowledge structure and the mechanisms of disease development, providing a deeper insight into the role played by mitochondria in ALI/ARDS and assisting in the discovery of ground-breaking strategies to clinical prevention and treatment problems. Therefore, this study systematically analyzes the scientific findings of mitochondria in ALI/ARDS, expecting to find new answers for the prevention and treatment of ALI/ARDS.

## 2. Materials and Methods

### 2.1. Data Collection

**Figure 1:**
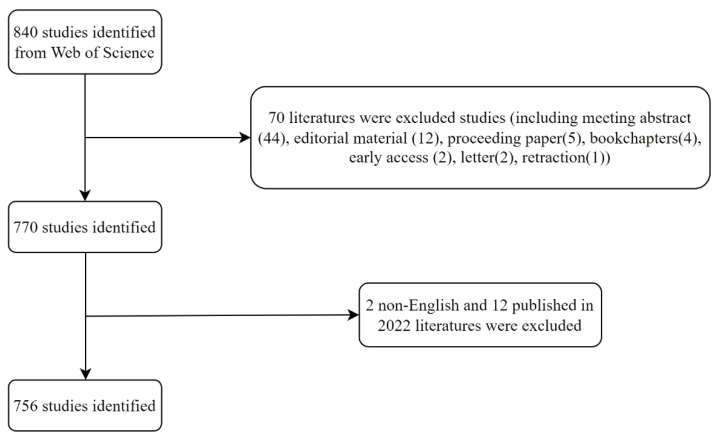
Flowchart of the screening process.

Our raw data were retrieved and downloaded from the Web of Science Core Collection (WoSCC) Expanded Science Citation Index (SCIE), developed by Thomson Scientific. Web of Science consists of a large number of high-quality and high-impact scientific studies, making it the most comprehensive and inclusive collection of information available worldwide. Due to the differences in citation data in each database, no database used for bibliometric studies is considered to be the most superior [25,26]. We chose WoSCC because it contains a comprehensive citation index record that offers a wide range of possibilities for bibliometric analysis, and it is the most commonly accepted database for bibliometric research at present [27,28]. Under the MeSH citation, the search terms were set as follows: TS = (mitochondria OR mitochondrion OR mitochondrial) AND TS = ((acute lung injury) OR (acute respiratory distress syndrome) OR (ALI) OR (ARDS) OR (respiratory distress syndrome)). We retrieved all articles and reviews in English on mitochondria in ALI/ARDS published online between 2012 and 2021 from the WoSCC’s SCIE. Meanwhile, to reduce bias, it was ensured that two observers conducted the literature search independently on the same date (30 September 2022) with a consistency of more than 0.9, indicating relative confidence [29]. Ultimately, 756 original articles and reviews were included in the analysis and further analyzed and visualized. The literature search process is shown in Figure 1.

### 2.2. Bibliometric Analysis

In this study, both objective and evaluative bibliometrics were used to visualize and analyze mitochondrial research findings in the field of ALI/ARDS. Objective bibliometrics measures the amount of literature, the number of citations and the analysis of citations [30]. The productivity, impact and quality of publications are expressed as the number of publications (Np), the number of citations (excluding self-citations) (Nc) and the average number of citations (Na) as Nc/Np, respectively. Evaluative bibliometrics measures the quantitative evaluation of the contributions of countries, authors, journals and institutions in the field and their quantifiable indices, such as the H-index [31,32]. The articles that influence the history of a field and show the current research hotspots as well as future trends can be identified by this analysis [33,34]. More importantly, the impact factor (IF) and local citation score (LCS) of the latest edition of the Journal Citation Reports (JCR) also indicate the value of an article [35,36]. In addition, the study topics were identified thanks to the easy-to-use data analysis capabilities of the WoSCC database, followed by the use of Endnote X9 and the R4.1.1-based Bibliometrix Package to store, clean and count the data.

VOSviewer, CiteSpace and R (version 4.1.3) were used for statistical calculations and graphical visualization. VOSviewer is a free Java-based software. The software’s clustering algorithm is related strength-based and provides a comprehensive and detailed bibliometric map based on collaborative data [37]. CiteSpace is software for analyzing the underlying knowledge contained in the scientific literature and visualizing the gathered data [38,39]. The software can visualize a field’s research results and predict future research trends by mapping the co-citation network of the literature [40]. R software (version 4.1.3) is a language environment widely applied to statistical computing and plotting. [41]. The bibliometrix package 3.2.1 in R was used for data cleaning, such as writing formatting, synonym merging and performing fundamental bibliometric analysis [42].

## 3. Results

### 3.1. The Global Overview of Publications

A total of 756 articles (580) and review articles (176) published within the last decade were retrieved. The total Nc of the retrieved publications was 20,762, the mean Nc per paper was 28.1 and the total H-index was 63. Overall, for mitochondrial-related research in the ALI/ARDS field, 4574 authors affiliated with 1217 institutions have been published in 346 journals worldwide, with authors from 61 countries or regions.

### 3.2. The Annual Trend of Publication Quantity

**Figure 2:**
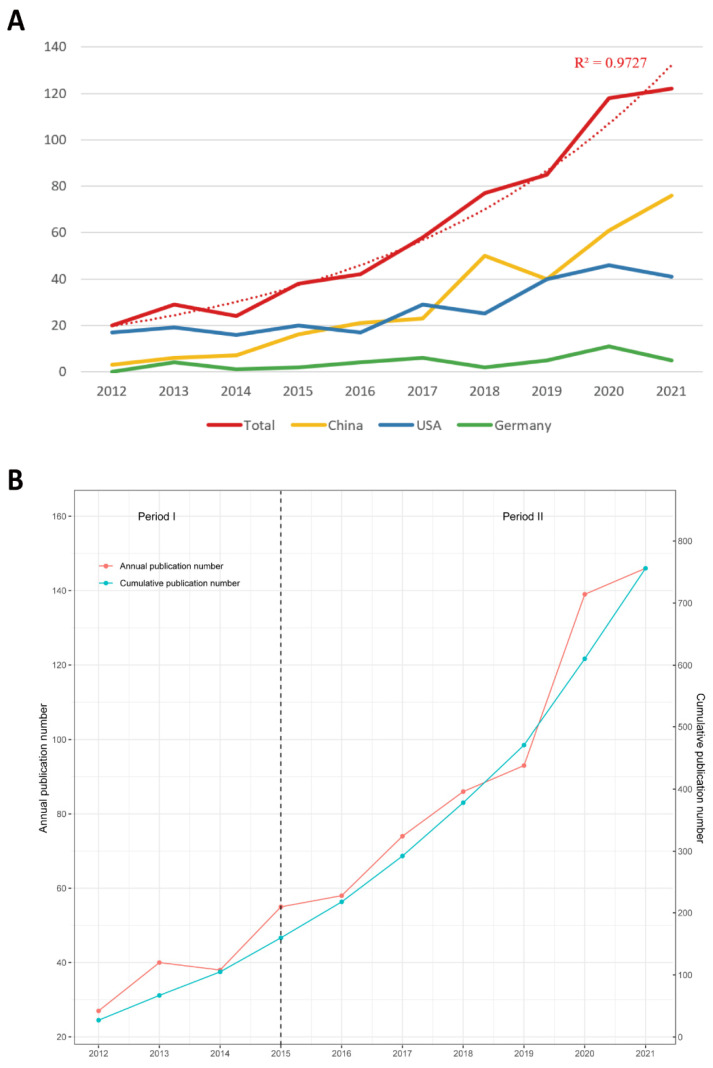
(**A**) The total numbers of publications and top three countries from 2012 to 2021. (**B**) The numbers of publications by year and accumulation from 2012 to 2021.

Figure 2A shows the annual Np related to mitochondria in ALI/ARDS. The total number of annual papers fluctuated between the decades but increased from 20 in 2012 to 122 in 2021 and showed an increasing trend. The polynomial fitting curve (cubic) with a correlation coefficient R^2^ of 0.9727 indicated a high correlation between the number of publications per year and the year of publication. The annual growth rate calculated using bibliometric methods was 35.7%, and the growth rate increased with time. At the level of the top three countries in terms of Np, the annual Np of China has increased rapidly since 2017, while the growth trends tend to be stable in the USA and Germany, indicating that China has been productive in this field of research in recent years.

In Figure 2B, the annual Np was divided into two phases. Using the research development model [43], we found that publication production was at a low level from 2012–2015 (Phase I). In this nascent phase, the relevant theories in the field were not entirely validated, and the focus on mitochondria in the field of ALI/ARDS was coming into the spotlight. From 2015 to the present (Phase II), the publication output in this field has shown a rapid increase, indicating that an increasing number of scholars are focusing on this field and conducting research, resulting in more research results. In summary, these findings suggest that the study of mitochondria in ALI/ARDS has become a hot topic and entered a phase of rapid development.

### 3.3. Analysis of Countries and Institutions

**Figure 3:**
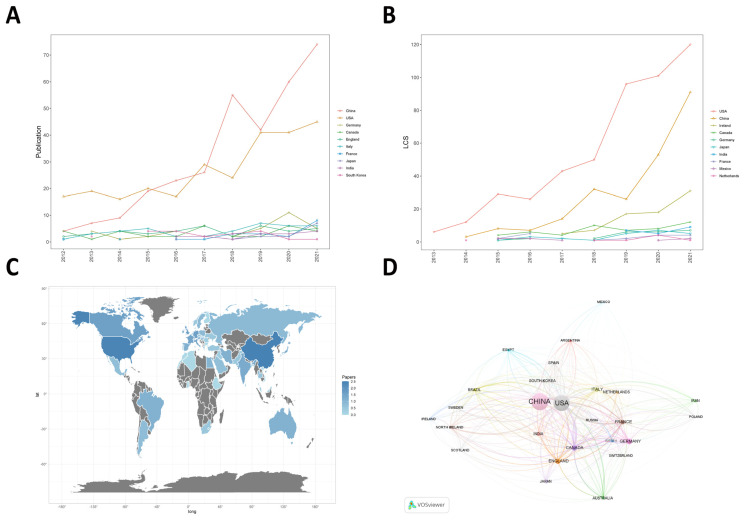
(**A**) Publications in the 10 most productive countries/regions. (**B**) The yearly number of local citation scores (LCS) of the 10 most productive countries/regions. (**C**) The country/region distribution of publications. Note: The blue shade indicates the number of publications, with darker blue indicating more publications. (**D**) The cooperation map of countries involving mitochondria in ALI. Note: The thicker the link between countries, the stronger the collaborative relationship, and vice versa.

A total of 61 countries/regions have published academic articles in the field of mitochondria in ALI/ARDS, but more than 75% of the publications were contributed by researchers from the top two most-active countries (China and the USA). We listed the top 10 countries with the highest publication output of articles (Table 1). China is the most published country in this field (327 papers/42.58%, 6217 citations, Na = 19.01), followed by the USA (270 papers/35.16%, 9816 citations, Na = 36.36), Germany, Canada, England and Italy. In terms of the H-index, China and the USA scored the highest, with 41 and 48, respectively. Compared to Germany, with an H-index of 17, and England, with an H-index of 19, Canada and Italy received higher scores of 24 and 21, respectively. As shown in Figure 3A, the Np levels were relatively low and stable in all countries except China and the USA. In 2021, although China ranks first in Np, the LCS is far below the USA (Figure 3B). This showed that while China had the highest Np in this field, the USA still led in terms of article quality, indicating that the USA had the greatest impact in this field. Figure 3C shows the level and distribution of Np in each country through the shades of colors. Figure 3D shows the cooperation relationship between different countries/regions. The connecting line indicates that two countries/regions cooperate, and the thicker the line is, the closer the cooperative relationship.

Table 2 lists the top 10 institutions with the highest Np of mitochondria in ALI/ARDS. The top three institutions with the highest Np were the University of California System, US Department of Veterans Affairs and Veterans Health Administration VHA, and, surprisingly, these three institutions not only had the highest Np (29, 27, and 26, respectively) but also had high H-index (17, 15, and 14, respectively). Although the Np of Harvard University in the USA was only 19, its Nc reached 1273 and Na reached 67, and, similarly, it reached the highest H-index of 18. Among the top 10 institutions, as many as six were in the USA, indicating that US institutions were more influential in this field, publishing many high-quality articles. In addition, Shanghai Jiao Tong University, Sichuan University and Chang Gung University, located in China, have also made some achievements in this field.

### 3.4. Analysis of Journals and Disciplines

A total of 346 journals published papers in the area of mitochondria and ALI/ARDS. Table 3 shows the overall profile of the top 10 journals in Np, including the H-index and the JCR-issued IF for 2021. Since these journals have published the most papers on related topics in the last decade, scholars in this research area could focus on these journals, and similarly, these journals were more likely to publish articles about mitochondria in ALI/ARDS. In addition, the average IF of these journals was upward of six, indicating that mitochondria in ALI/ARDS was a promising research field favored by high-level journals and deserved further exploration.

**Figure 4:**
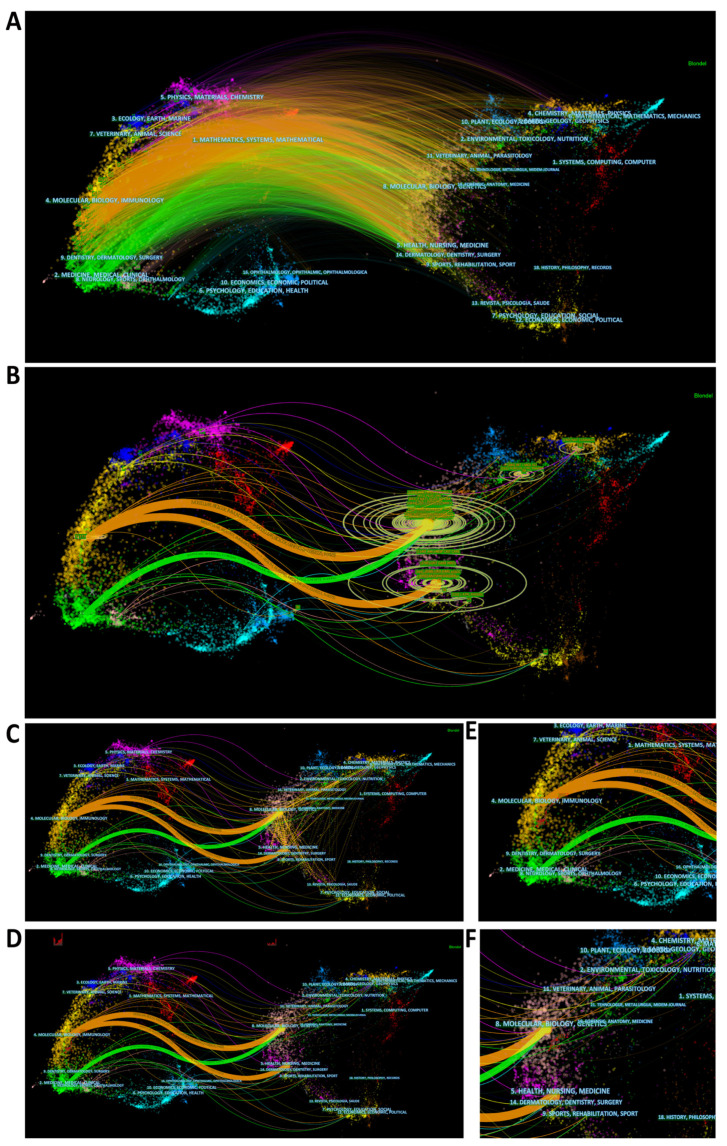
(**A**) The dual-map overlay of citing and cited article. Labels indicate corresponding disciplines of citing or cited articles. (**B**) The dual-map overlay using z-score function. (**C**) The co-citation links of the cited journals. (**D**) The citing and cited trajectory from 2012 to 2021. (**E**,**F**) The partial enlargement of the trajectory. Note: Different colors represent different discipline categories. The thicker the connection between disciplines, the stronger the citation relationship, and vice versa.

The dual-map overlay obtained using CiteSpace is a useful tool to show the disciplines to which they belong. The left and right sides of the dual-map overlay represent citing and cited journals, respectively, and are interconnected by citation waves. The citing side can be considered the research frontier, and the cited side can be considered the basis of their research [44,45]. The labels at the center of the clustering indicate the corresponding disciplines of the citing or cited articles (Figure 4A). The z-score function allows for highlighting strong connections, making them easier to identify (Figure 4B). Journals with larger numbers are marked with circles of different sizes at the end of the citation wave. In this overlay, the largest and most dominant source disciplines were easily identified as (1) “Molecular, Biology, Immunology”. (2) “Veterinary, Animal, Science”, (3) “Medicine, Medical, Clinical” and (4) “Physics, Materials, Chemistry”. Most of the citations linked point to two disciplines: (1) “Molecular, Biology, Genetics” and (2) “Health, Nursing, Medicine”. In addition, (3) “Environmental, Toxicology, Nutrition” and (4) “Chemistry, Materials, Physics” were also attractive. The dashed lines across the different clusters in Figure 4C indicate the co-citation connections of the cited journals, from which we can see intense cross-border connections between the four most cited major disciplines. Figure 4D–F shows the citing and cited path starting and ending the year on the corresponding side, whose trajectory can help illustrate the dynamics of publication at the disciplinary level [45]. The citing trajectory presents a general shift from lower zones to upper areas on the map. Based on the citation waves, the starting point of the citing trajectory appears to be influenced by the development of the discipline of “Medicine, Medical, Clinical”. In contrast, the ending point of the citing trajectory is significantly dominated by publications in the field of “Molecular, Biology, Immunology”. This shows a rough tendency for the hotspots of the research front to drift from clinical to basic research over the past ten years.

### 3.5. Analysis of Local Citation Score (LCS)

**Figure 5:**
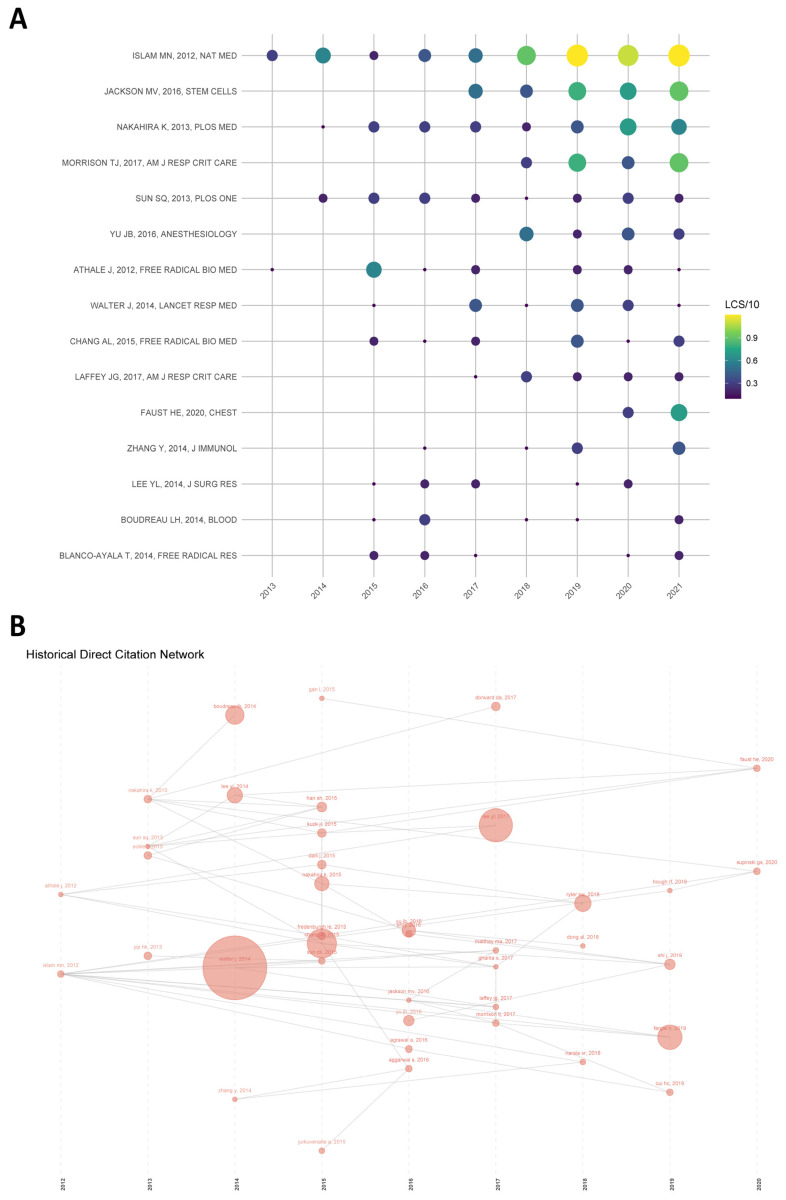
(**A**) The yearly number of local citations of papers with high local citation scores (LCS). Note: The size and colors of the circle represent the LCS of papers in that year. The larger the size and the more yellow the color of the circle, the higher the LCS is. (**B**) One paper cited by the other papers with the top 40 LCS. Note: Each node in the figure signifies a key article, and the directional arrow specifies the citation association between the two articles. The size of each node is proportional to the frequency of this article by the other 39 articles.

The LCS significantly represents the impact of an article in the local field. Figure 5A shows the details of the top 15 articles in terms of local citations obtained from our LCS analysis. Figure 5B shows a network diagram of the top 40 papers in the LCS rankings, with each node representing a cited paper. Each node is sized based on the frequency of this article among the other 39 articles. Unsurprisingly, an article published by Islam MN et al. [46] in Nature Medicine in 2012 obtained the highest LCS score. This study showed that mitochondrial transfer from bone marrow mesenchymal stem cells (BMSCs) to the alveoli effectively prevents ALI, laying the foundation for research on the protective role of stem cells by transferring mitochondria. In addition, an article published by Jackson MV et al. [47] in Stem Cells in 2016 also received a high LCS score, which demonstrated for the first time that mitochondrial transfer from MSC to macrophages can lead to their enhanced phagocytosis and revealed an important new mechanism for the protective role of MSC in the lung. Similarly, Morrison TJ et al. [48] published an article in American Journal of Respiratory and Critical Care Medicine in 2017 corroborating that MSC can regulate macrophages in clinically relevant lung injury models through extracellular vesicular mitochondrial transfer. Thus, the treatment of ALI by stem cell mitochondrial transfer has become a recent hot topic for researchers in this field, and many results have been achieved that have had a significant impact. Except for Faust He et al. [49], who published an article on plasma mtDNA levels in trauma and sepsis patients with ARDS in 2020, the remaining 14 studies were conducted between 2012 and 2017, indicating that research in the field was in a primary stage during this period, while scholars around the world have continued to build on these classic studies to expand the impact of the field after 2017.

### 3.6. Analysis of Hotspots and Frontiers

**Figure 6:**
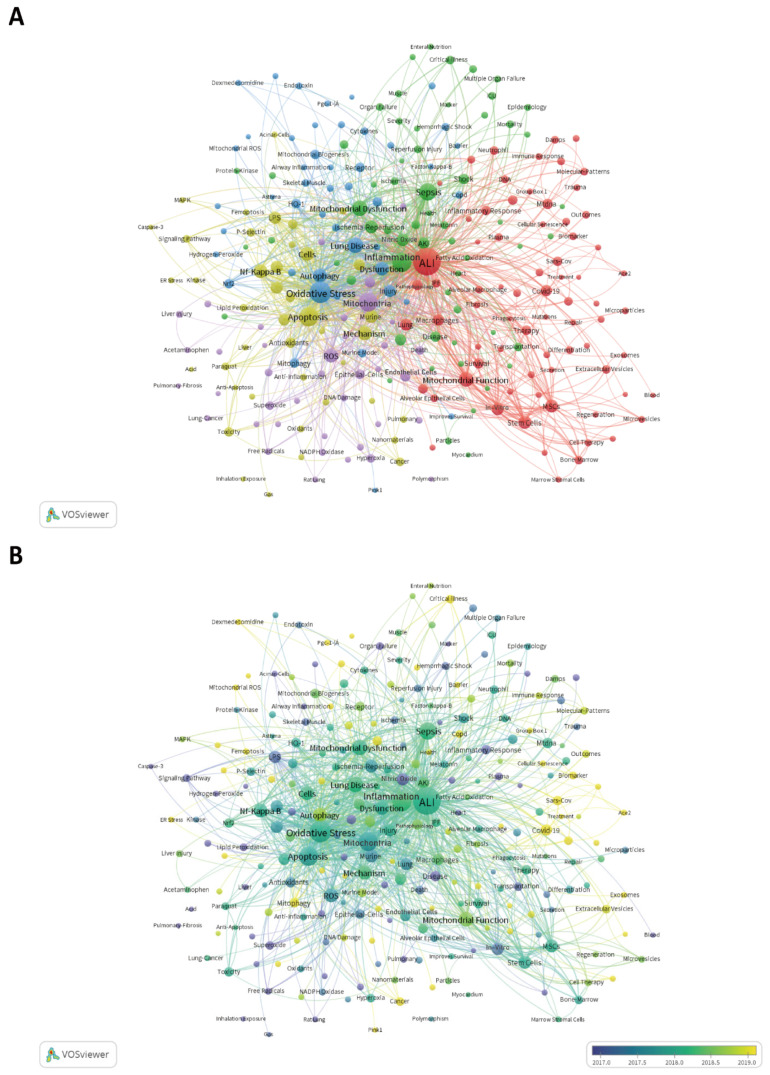
The mapping on keywords of mitochondria in ALI. (**A**) The 260 keywords that occurred more than five times were divided into five clusters by different colors: cluster 1: red, cluster 2: green, cluster 3: blue, cluster 4: yellow, cluster 5: purple. Note: The size of the node represents the frequency of occurrence. The color of the node represents the different clusters. The thickness of the lines represents the link strength. (**B**) Visualization of keywords according to the APY. Note: The size of the node represents the frequency of occurrence. The color of the node represents the time when the keyword appeared. Keywords in yellow appeared later than that in blue.

Keyword co-occurrence analysis easily summarizes the themes of various studies in a field and suggests research hotspots and directions. We visualized the hot themes in this field using VOSviewer (Figure 6A). The 260 keywords that appeared more than five times were divided into five clusters according to different colors: Cluster 1 (red): mechanisms and pathways of effects on mitochondria in the prevention and treatment of ALI/ARDS; Cluster 2 (green): relationships between mitochondrial metabolism and sepsis and inflammation; Cluster 3 (blue): mitophagy and NLPR3 inflammasome activation pathways; Cluster 4 (yellow): mechanisms of effects between mitochondria and lipopolysaccharide (LPS)-induced apoptosis in ALI/ARDS; and Cluster 5 (purple): relationships between mitochondria and reactive oxygen species (ROS)-induced oxidative stress with lung epithelial and lung endothelial cells. The size of the nodes represents the frequency of occurrence. The most frequent keywords in addition to “ALI” and “mitochondria” were “oxidative stress”, “inflammation”, “apoptosis”, “sepsis” and “autophagy”, indicating that basic research related to mitochondria in ALI/ARDS was fruitful in terms of cell death mechanisms. In addition, Figure 6B shows the visualization of the keywords according to APY. The yellow keywords appeared later than the blue ones. The latest keywords were COVID-19 (Cluster 1, APY: 2020) and SARS-CoV (Cluster 1, APY: 2020), which were closely related to the outbreak of the COVID-19 epidemic in the last two years.

### 3.7. Analysis of Co-Cited Reference Clusters

**Figure 7:**
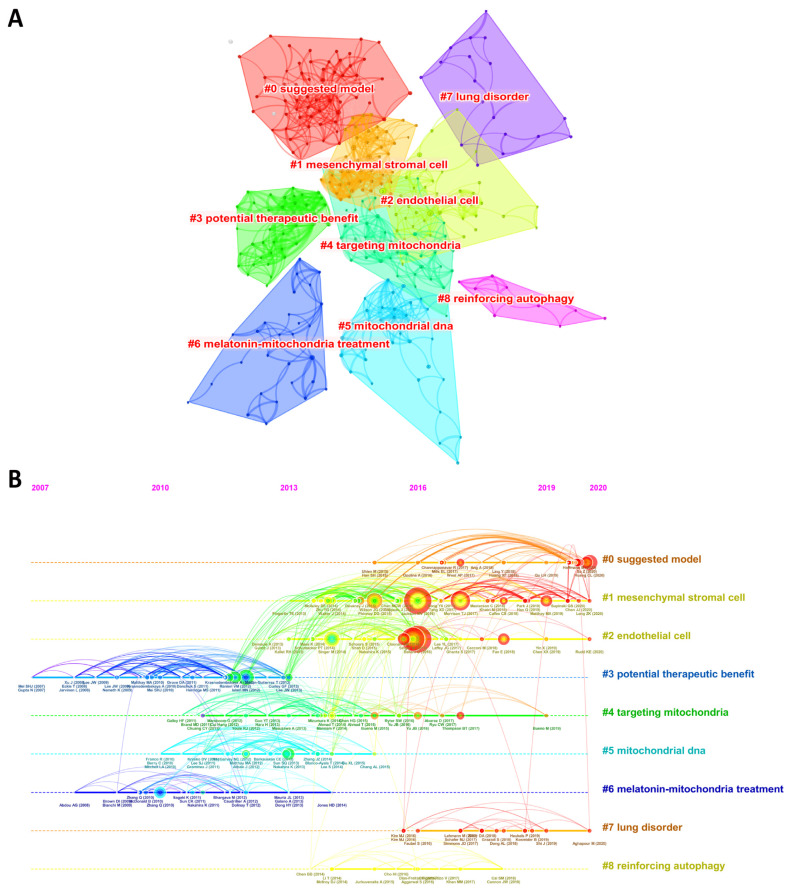
(**A**) The clustered network map of co-cited references related to mitochondria in ALI. Note: Each node represents a reference, and different colors represent different clusters. (**B**) The timeline view of co-citation clusters with their cluster-labels on the right. Note: Each node represents a reference, the size of the circle represents the citation frequency, and the red color of the circles and the connecting curves represents the latest year.

Co-citation networks can help scholars understand the important publications, research bases, and research hotspots and frontiers of a field. Therefore, we used the CiteSpace tool to classify these references into nine clusters: #0 suggested model, #1 mesenchymal stromal cell, #2 potential therapeutic benefit, #3 endothelial cell, #4 targeting mitochondria, #5 mitochondrial DNA, #6 melatonin-mitochondria treatment, #7 lung disorder, and #8 reinforcing autophagy. The silhouette value of each cluster was greater than 0.8, and the numbers 0 to 8 represented the clusters from large to small. Figure 7A visualizes the above clusters. Figure 7B shows a timeline view of the co-citation clusters, with the annual rings representing the frequency of citations, the thickness of the rings representing the number of citations, and the connections between the nodes indicating the co-citation intensity, from purple to yellow representing the years of publication from 2007 to 2021. The results showed that #1 mesenchymal stromal cell and #3 endothelial cell had the most citation bursts. In addition, research hotspots have shifted from potential therapeutic treatments and mtDNA to endothelial cell and MSC.

### 3.8. Burst Detection

**Figure 8:**
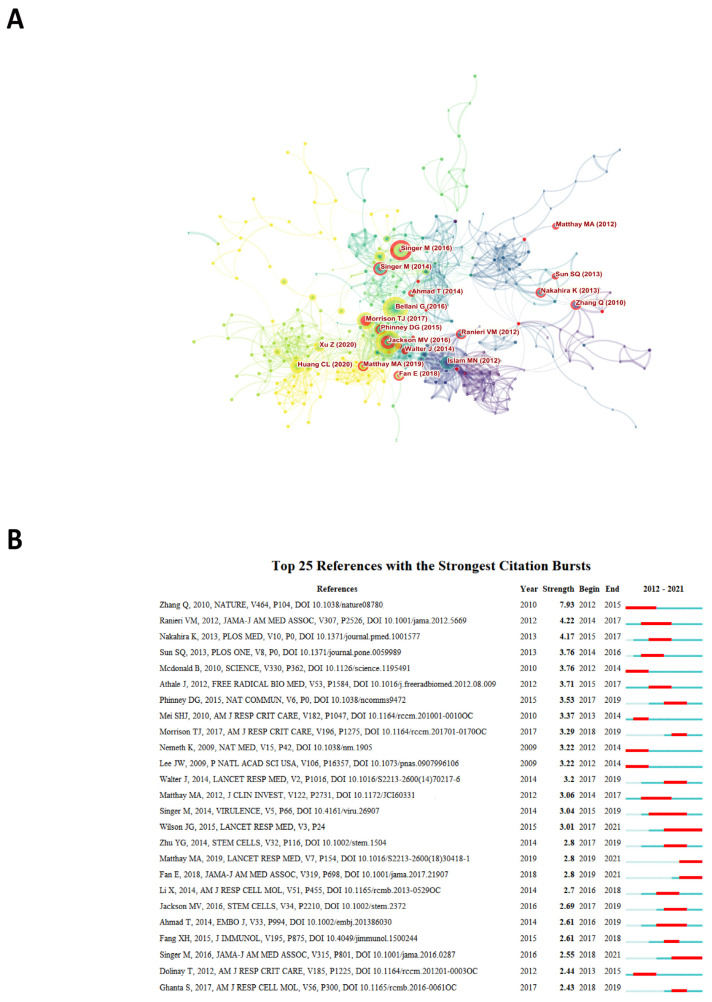
(**A**) Papers with the strongest citation bursts in original articles on the mitochondria in ALI between 2012 and 2021. Note: Each node represents a cited reference, and the red circle outside the node means that the reference has a strong citation burst. The links between nodes reflect the strength of the co-citation. The different colors represent the publication year of the paper, with purple being the earliest and yellow being the latest. (**B**) The top 25 references with the strongest citation bursts.

A citation burst is a sudden increase in the number of citations during a short period, and burst detection reveals the most active areas and emerging trends in the network. Figure 8A shows the papers with the strongest citation bursts in research on mitochondria in ALI/ARDS between 2012 and 2021. Each node represents a cited reference, and the red circle outside the node means that the reference has a strong burst. Interestingly, we can see that the three studies that ranked high in the LCS, from Islam MN [46], Jackson MV [47], and Morrison TJ [48], also appeared in the citation blast, thus showing that these three studies have had a great and far-reaching impact. Figure 8B then shows the details of the top 25 references with the highest citations according to citation strength.

## 4. Discussion

In this study, we collected data from 756 papers on mitochondria in ALI/ARDS during the last decade from the SCIE database. We performed a series of bibliometric analyses and data visualization through the Biblimetrix (3.1.4) R package, CiteSpace (5.8. R3) and VOS viewer, revealing that the number of publications in this field per year shows a rapid growth trend, especially after 2015.

Among the top-ranked countries/regions, China ranked first in production (327, 42.58%), followed by the USA (270, 35.16%), indicating that China and the USA led in this field. In recent years, China’s scientific development has grown by leaps and bounds. China’s position as the largest publishing country was closely related to its substantial support for scientific research and the vigorous training of scientific talent. However, compared to China, the USA had higher Nc, Na and H indices, representing a more solid research foundation and results in this field. More importantly, six of the top 10 affiliates were from the USA, which may be one of the reasons why the USA was more influential in mitochondrial research in ALI/ARDS. Except for China and the USA, the publication volume was low, but Canada, England, Italy, France and Korea were at a high level of Na index, representing the relatively high quality of publications in these countries. In this regard, scholars and affiliates in China, Germany, Japan and India should make further efforts to improve the quality of papers in this area. As seen from the co-occurrence network, China and the USA take a central position with the thickest line between them, which represents a very close collaborative relationship. In addition, the connecting lines between countries are intertwined, indicating that scholars and institutions in each country contribute their respective strengths to remove academic barriers and promote academic cooperation and exchange.

Of the top 10 journals with the highest Np, seven had an IF above 5, indicating a high demand for research exploration of mitochondria in ALI/ARDS and that most of the research in this field was of high quality. It is noteworthy that almost all of the top 10 articles of LCS were published in very high IF journals, including three articles on the mitochondrial transfer of stem cells, which created a great impact by establishing the basis of research in this direction. In addition, based on the burst detection findings, it was suggested that stem cells and endothelial cells were a recent research hotspot in this field; therefore, scholars in this field can actively focus on these topics. As seen from the double map overlay, the main disciplines at the forefront of research have undergone a general shift from “Medicine, Medical, Clinical” to “Molecular, Biology, Immunology” over the past decade, meaning that the direction of research in this field has gradually shifted from clinical research to basic research. “Molecular, Biology, Immunology” is still the most important research discipline in general. In addition, the two cited disciplines of “Molecular, Biology, Genetics” and “Health, Nursing, Medicine” have been closely related for many years. The results suggest that research on mitochondria in ALI/ARDS is currently focused on molecular, biological, immunological and genetic science; thus, scholars in this field should consider delving deeper into these disciplines to discover more exhaustive mechanisms.

We found that the years from 2012 to 2015 were the nascent phase of the field, and the research in this phase was mainly focused on establishing the theoretical foundations for later studies. For example, Islam MN et al. [46] discovered that mitochondria derived from BMSCs could be transferred into the alveoli and thus prevent the development of ALI, which laid a considerable research foundation for the role of stem cell mitochondrial transfer in ALI, and later scholars continued to build on this research with further innovations. In addition, Athale J et al. [50] found that Nrf2 deficiency inhibited mitochondrial biogenesis and anti-inflammatory communication in the alveoli, thereby exacerbating ALI. This study links redox activation of mitochondrial biogenesis to the treatment of ALI. The dramatic increase in ROS can lead to an imbalance in mitochondrial fusion/division, which largely accelerates the progression of sepsis-associated multiorgan failure [51]. In septic ALI, massive expression of the inflammatory amplification factor Trem-1 specifically increased Mfn-1 and Mfn-2 expression in macrophages, which contributed to the maintenance of mitochondrial integrity in macrophages [52]. CHANG AL et al. [53] found that mitophagy in the alveolar region was dependent on the activation of Nrf2 and that promoting mitophagy may be an effective treatment for septic ALI. Zhang Y et al. [54] found that deletion of the PINK1 gene could increase the susceptibility of lung endothelial cells to hyperoxia through mitophagy. mtDNA is a potent inflammatory trigger that can be present extracellularly in the blood in pathological states. A clinical study found for the first time that elevated mtDNA levels were associated with ICU mortality; therefore, mtDNA could be used as a plasma biomarker for risk prediction in ICU patients [55], which also provided clinical evidence for later basic research on mtDNA in ALI. In addition, Lee YL et al. [56] found that blood transfusion products contain molecular patterns associated with mtDNA damage, which may be a potential effector of transfusion-associated ALI. The study by Boudreau LH et al. determined that extracellular mitochondria were produced by platelets and played an important role in promoting the inflammatory response [57]. During this phase, there was a burst of research on the potential therapeutic benefits of mitochondria as well as mtDNA, which can also be seen on the timeline in Figure 7B.

In the next phase of rapid publication growth, scholars in the field focused on the specific mechanisms and signaling pathways of mitochondria in ALI/ARDS and considered the role of mitochondria in various lung diseases and cellular interactions. Mitophagy, mitochondrial biogenesis, mitochondrial energy metabolism, cellular autophagy and apoptosis are gradually being unveiled by researchers in this field. Signaling molecules and pathways, such as ROS, NLRP3, Nrf2, HO-1, and NF-κB, have also been tightly linked to mitochondrial functions. For instance, Yu J et al. [58] found that the HO-1/carbon monoxide system ameliorated the imbalance of LPS-induced dynamic mitochondrial fusion/fission processes, effectively attenuating LPS-induced ALI. In addition, significant breakthroughs have been made in the mitochondrial transfer of stem cells. Jackson MV et al. [47] showed that a large number of mitochondria could be transferred from MSC to macrophages by tunneling nanotube (TNT)-like structures, and macrophages that were able to phagocytose MSC-derived mitochondria showed enhanced phagocytic activity. This study demonstrated for the first time that mitochondrial transfer of MSC can lead to enhanced phagocytic activity of innate immune cells and revealed an important new mechanism for the antimicrobial role of MSC in ARDS. Morrison TJ et al. [48] also showed that MSC ameliorates ALI by promoting an anti-inflammatory macrophage phenotype through EV-mediated mitochondrial translocation, the process of which was very dependent on enhanced oxidative phosphorylation within macrophages. Laffey JG [59] also detailed in a review that MSC exerts anti-inflammatory and pro-lysis effects on injured lung endothelium and alveolar epithelium through the release of paracrine factors, microvesicles and mitochondrial translocation. A clinical study showed a further indication of the potential correlation between circulating mtDNA and lung injury, which deserves further investigation as a targeted mediator of ARDS [49]. The five studies mentioned above also received high LCS scores due to the great impact in their respective fields, which is reflected in Figure 5A. Furthermore, based on in vitro and in vivo studies, mitochondrial oxidative stress played a key role in endothelial barrier regulation of ALI, and inflammation-mediated lung injury in diverse ALI models can be mitigated through the use of mitochondrial antioxidants (MitoTEMPO) [60]. Similarly, increased levels of mitochondrial oxidative stress were found to correspond to disease severity in ICU patients with ALI/ARDS [61].

With the outbreak of the COVID-19 epidemic, researchers began to explore the relationship between mitochondria and COVID-19. It has been shown that COVID-19 viruses can manipulate the host cytoplasm to release mtDNA and activate the mtDNA-induced inflammasome, thereby suppressing innate and adaptive immunity [62]. Mitochondrial oxidative stress may contribute to microbiota dysbiosis, fostering an inflammatory/oxidative response that leads to a vicious cycle of events [63]. ORF-9b is a protein virulence factor of SARS-CoV-2 that is located in mitochondria and leads to excessive fusion of mitochondria by triggering ubiquitin and proteasome degradation of DRP-1 [64], which is a mechanism involved in resistance to apoptosis [65]. These findings suggest that the differential localization of viral RNA and proteins in mitochondria play a crucial role in the pathogenesis of SARS-CoV-2. The majority of patients with severe COVID-19 are elderly individuals, who are more susceptible to death from infections and complications of chronic diseases because of the age-related decline in mitochondrial function that leads to chronic metabolic disorders such as diabetes or cancer. Understanding the mechanisms of the COVID-19 virus–host mitochondrial interaction could provide new approaches to the prevention and treatment of different causes of ALI/ARDS, including COVID-19, which still seems to be nonnegligible.

The VOS Viewer, CiteSpace and R (Version 4.1.3) allow a visual analysis of the papers, thus revealing research trends and hotspots in the field. Meanwhile, through LCS and burst detection, we found some essential classical papers that have been the basis of research in the field, leading the development direction, exercising great influence and providing further research ideas. Compared to traditional reviews, bibliometric studies provide more visual analysis and better tracking of changing research hotspots. Moreover, this study utilizes more visualization tools than some past bibliometric studies to present the results of research in a field from multiple perspectives. However, there are still some limitations. First, the data in this study only included articles in English and reviews from SCI-Expanded. Second, we only included papers from 2012–2021. Ten years, as a common time span for bibliometric analysis, provides a great overview of the developmental history of the field but may overlook earlier classical research. In addition, we excluded influential low-NC papers published in 2022, making this study unavoidably delayed. Therefore, a bibliometric analysis of newly published articles should be continued at specific times to enrich the findings in this area continuously.

## 5. Conclusions

This bibliometric analysis reveals the research directions and frontier hotspots of mitochondria in ALI/ARDS, which has shown a rapid growth trend in annual publication numbers. China and the USA have led the field, with China producing the largest number of papers and the USA producing higher-quality research. Similarly, many of the classical theories came from the research of USA institutions. The major disciplines at the forefront of research have shifted from clinical research to basic research. mtDNA, mitophagy and apoptosis have been the most active research areas, while studies on mitochondrial transfer in stem cells have become a hot topic in recent years. In addition, studies on the mechanisms of mitochondria in COVID-19-associated ALI/ARDS are still of great potential value.

## Figures and Tables

**Table 1:** Publications in the 10 most productive countries/regions.

Rank	Country/Region	Np	% of (756)	Nc	Na	H-Index
1	China	327	42.58	6217	19.01	41
2	USA	270	35.16	9816	36.36	48
3	Germany	40	5.21	999	24.98	17
4	Canada	39	5.08	2019	51.77	24
5	England	35	4.56	1694	48.40	19
6	Italy	33	4.30	1148	34.79	21
7	France	29	3.78	977	33.62	16
8	Japan	21	2.73	486	23.14	13
9	India	20	2.60	338	16.90	8
10	South Korea	17	2.21	679	39.94	13

**Table 2:** The top 10 productive institutions.

Rank	Institutions	Country	Np	Nc	Na	H-Index
1	University of California System	USA	29	1345	46.38	17
2	US Department of Veterans Affairs	USA	27	837	31.00	15
3	Veterans Health Administration VHA	USA	26	812	31.23	14
4	Shanghai Jiao Tong University	China	24	461	19.21	12
5	Pennsylvania Commonwealth System of Higher Education PCSHE	USA	20	713	35.65	11
6	Harvard University	USA	19	1273	67	18
7	Sichuan University	China	19	297	15.63	11
8	University of Pittsburgh	USA	19	706	37.16	11
9	Chang Gung University	China	18	660	36.67	11
10	University of Toronto	Canada	18	1204	66.89	15

**Table 3:** The top 10 productive journals.

Rank	Journal	Np	Nc	Na	H-Index	IF (2021)
1	Am. J. Physiol.-Lung C	28	736	26.29	17	6.011
2	Free Radical. Bio. Med.	20	759	37.95	15	8.101
3	Int. J. Mol. Sci.	20	451	22.55	10	6.208
4	Oxid Med. Cell Longev.	19	402	21.16	13	7.310
5	PLoS ONE	15	504	33.60	12	3.752
6	Front Immunol.	14	563	40.21	9	8.786
7	Shock	13	229	17.62	10	3.533
8	Sci. Rep.-Uk	11	157	14.27	7	4.996
9	Am. J. Resp. Cell Mol.	10	225	22.50	9	7.748
10	Biomed. Pharmacother.	9	110	12.22	6	7.419

## Data Availability

The datasets generated and/or analyzed during the current study are available in the [The Science Citation Index Expanded (SCIE 1975–present) of Clarivate Analytics’ Web of Science Core Collection (WoSCC)] repository, https://www.webofscience.com/wos/alldb/basic-search, accessed on, accessed on 30 September 2022.

## Abbreviations

ALI: acute lung injury; ARDS: acute respiratory distress syndrome; SCIE: Science Citation Index Expanded; WoSCC: Web of Science Core Collection; mtDNA: mitochondrial DNA; MSC: mesenchymal stromal cell; ICU: intensive care unit; mtROS: mitochondrial ROS; Np: number of publications; Nc: number of citations (excluding self-citations); Na: average number of citations; IF: impact factor; LCS: local citation score; JCR: Journal Citation Reports; BMSCs: bone marrow mesenchymal stem cells; LPS: lipopolysaccharide; ROS: reactive oxygen species; TNT: tunneling nanotube; MitoTEMPO: mitochondrial antioxidants.

## References

[1] Shah F.A., Meyer N.J., Angus D.C., Awdish R., Azoulay É., Calfee C.S., Clermont G., Gordon A.C., Kwizera A., Leligdowicz A. (2021). A Research Agenda for Precision Medicine in Sepsis and Acute Respiratory Distress Syndrome: An Official American Thoracic Society Research Statement. Am. J. Respir. Crit. Care Med..

[2] Ranieri V.M., Rubenfeld G.D., Thompson B.T., Ferguson N.D., Caldwell E., Fan E., Camporota L., Slutsky A.S., ARDS Definition of Task Force (2012). Acute Respiratory Distress Syndrome: The Berlin Definition. JAMA.

[3] Bellani G., Laffey J.G., Pham T., Fan E., Brochard L., Esteban A., Gattinoni L., Van Haren F., Larsson A., McAuley D.F. (2016). Epidemiology, Patterns of Care, and Mortality for Patients with Acute Respiratory Distress Syndrome in Intensive Care Units in 50 Countries. JAMA.

[4] Meyer N.J., Gattinoni L., Calfee C.S. (2021). Acute Respiratory Distress Syndrome. Lancet.

[5] Matthay M.A., Zemans R.L., Zimmerman G.A., Arabi Y.M., Beitler J.R., Mercat A., Herridge M., Randolph A.G., Calfee C.S. (2019). Acute Respiratory Distress Syndrome. Nat. Rev. Dis. Prim..

[6] Pham T., Pesenti A., Bellani G., Rubenfeld G., Fan E., Bugedo G., Lorente J.A., Fernandes A.D.V., Van Haren F., Bruhn A. (2021). Outcome of Acute Hypoxaemic Respiratory Failure: Insights from the Lung Safe Study. Eur. Respir. J..

[7] Johnson E.R., Matthay M.A. (2010). Acute Lung Injury: Epidemiology, Pathogenesis, and Treatment. J. Aerosol. Med. Pulm. Drug Deliv..

[8] Choudhuri S., Chowdhury I.H., Garg N.J. (2020). Mitochondrial Regulation of Macrophage Response against Pathogens. Front. Immunol..

[9] Dumont A., Lee M., Barouillet T., Murphy A., Yvan-Charvet L. (2021). Mitochondria Orchestrate Macrophage Effector Functions in Atherosclerosis. Mol. Asp. Med..

[10] Artyomov M.N., Sergushichev A., Schilling J.D. (2016). Integrating Immunometabolism and Macrophage Diversity. Semin. Immunol..

[11] Ramond E., Jamet A., Coureuil M., Charbit A. (2019). Pivotal Role of Mitochondria in Macrophage Response to Bacterial Pathogens. Front. Immunol..

[12] Wang Y., Li N., Zhang X., Horng T. (2021). Mitochondrial Metabolism Regulates Macrophage Biology. J. Biol. Chem..

[13] Porporato P.E., Filigheddu N., Pedro J.M.B., Kroemer G., Galluzzi L. (2018). Mitochondrial Metabolism and Cancer. Cell. Res..

[14] Rensvold J.W., Shishkova E., Sverchkov Y., Miller I.J., Cetinkaya A., Pyle A., Manicki M., Brademan D.R., Alanay Y., Raiman J. (2022). Defining Mitochondrial Protein Functions through Deep Multiomic Profiling. Nature.

[15] Sharma A., Ahmad S., Ahmad T., Ali S., Syed M.A. (2021). Mitochondrial Dynamics and Mitophagy in Lung Disorders. Life Sci..

[16] Rowlands D.J. (2016). Mitochondria dysfunction: A novel therapeutic target in pathological lung remodeling or bystander?. Pharm. Ther..

[17] Lerner C.A., Sundar I.K., Rahman I. (2016). Mitochondrial Redox System, Dynamics, and Dysfunction in Lung Inflammaging and Copd. Int. J. Biochem. Cell. Biol..

[18] Khan M.S., Ullah W., Bin Riaz I., Bhulani N., Manning W.J., Tridandapani S., Khosa F. (2016). Top 100 Cited Articles in Cardiovascular Magnetic Resonance: A Bibliometric Analysis. J. Cardiovasc. Magn. Reason..

[19] Chen S., Zhang Y., Dai W., Qi S., Tian W., Gu X., Chen X., Yu W., Tian J., Su D. (2020). Publication Trends and Hot Spots in Postoperative Cognitive Dysfunction Research: A 20-Year Bibliometric Analysis. J. Clin. Anesth..

[20] Seriwala H.M., Khan M.S., Shuaib W., Shah S.R. (2015). Bibliometric Analysis of the Top 50 Cited Respiratory Articles. Expert Rev. Respir. Med..

[21] Huang X., Liu X., Shang Y., Qiao F., Chen G. (2020). Current Trends in Research on Bone Regeneration: A Bibliometric Analysis. Biomed. Res. Int..

[22] Qiu Y., Yang W., Wang Q., Yan S., Li B., Zhai X. (2018). Osteoporosis in Postmenopausal Women in This Decade: A Bibliometric Assessment of Current Research and Future Hotspots. Arch. Osteoporos..

[23] He J., He L., Geng B., Xia Y. (2021). Bibliometric Analysis of the Top-Cited Articles on Unicompartmental Knee Arthroplasty. J. Arthroplast..

[24] Shah S.M., Ahmad T., Chen S., Yuting G., Liu X., Yuan Y. (2021). A Bibliometric Analysis of the One Hundred Most Cited Studies in Psychosomatic Research. Psychother. Psychosom..

[25] Ogunsakin R.E., Ebenezer O., Ginindza T.G. (2022). A Bibliometric Analysis of the Literature on Norovirus Disease from 1991–2021. Int. J. Environ. Res. Public Health.

[26] Ogunsakin R.E., Ebenezer O., Jordaan M.A., Shapi M., Ginindza T.G. (2022). Mapping Scientific Productivity Trends and Hotspots in Remdesivir Research Publications: A Bibliometric Study from 2016 to 2021. Int. J. Environ. Res. Public Health.

[27] You Y., Li W., Liu J., Li X., Fu Y., Ma X. (2021). Bibliometric Review to Explore Emerging High-Intensity Interval Training in Health Promotion: A New Century Picture. Front Public Health.

[28] Igwaran A., Edoamodu C.E. (2021). Bibliometric Analysis on Tuberculosis and Tuberculosis-Related Research Trends in Africa: A Decade-Long Study. Antibiotics.

[29] Landis J.R., Koch G.G. (1977). The Measurement of Observer Agreement for Categorical Data. Biometrics.

[30] Wang S., Zhou H., Zheng L., Zhu W., Zhu L., Feng D., Wei J., Chen G., Jin X., Yang H. (2021). Global Trends in Research of Macrophages Associated with Acute Lung Injury over Past 10 Years: A Bibliometric Analysis. Front. Immunol..

[31] Li T., Yang A., Liu G., Zou S., Chen Y., Ni B., Liu Y., Fan J. (2021). Status Quo and Research Trends of Craniopharyngioma Research: A 10-Year Bibliometric Analyses (from 2011 to 2020). Front. Oncol..

[32] Hirsch J.E. (2005). An Index to Quantify an Individual’s Scientific Research Output. Proc. Natl. Acad. Sci. USA.

[33] Moed H.F. (2009). New Developments in the Use of Citation Analysis in Research Evaluation. Arch. Immunol. Ex.

[34] Brandt J.S., Hadaya O., Schuster M., Rosen T., Sauer M.V., Ananth C.V. (2019). A Bibliometric Analysis of Top-Cited Journal Articles in Obstetrics and Gynecology. JAMA Netw. Open.

[35] Jones T., Huggett S., Kamalski J. (2011). Finding a Way through the Scientific Literature: Indexes and Measures. World Neurosurg..

[36] Roldan-Valadez E., Salazar-Ruiz S.Y., Ibarra-Contreras R., Rios C. (2019). Current Concepts on Bibliometrics: A Brief Review About Impact Factor, Eigenfactor Score, Citescore, Scimago Journal Rank, Source-Normalised Impact Per Paper, H-Index, and Alternative Metrics. Ir. J. Med. Sci..

[37] Van Eck N.J., Waltman L. (2010). Software Survey: Vosviewer, a Computer Program for Bibliometric Mapping. Scientometrics.

[38] Yao L., Hui L., Yang Z., Chen X., Xiao A. (2020). Freshwater Microplastics Pollution: Detecting and Visualizing Emerging Trends Based on Citespace Ii. Chemosphere.

[39] Chen C. (2004). Searching for Intellectual Turning Points: Progressive Knowledge Domain Visualization. Proc. Natl. Acad. Sci. USA.

[40] Liu S., Sun Y.P., Gao X.L., Sui Y. (2019). Knowledge Domain and Emerging Trends in Alzheimer’s Disease: A Scientometric Review Based on Citespace Analysis. Neural. Regen. Res..

[41] Aria M., Cuccurullo C. (2017). Bibliometrix: An R-Tool for Comprehensive Science Mapping Analysis. J. Informetr..

[42] Hao X., Liu Y., Li X., Zheng J. (2019). Visualizing the History and Perspectives of Disaster Medicine: A Bibliometric Analysis. Disaster Med. Public Health Prep..

[43] Frame J.D., Baum J.J., Card M. (2014). An Information Approach to Examining Developments in an Energy Technology: Coal Gasification. J. Am. Soc. Inf. Sci. Technol..

[44] Chen C. (2017). Science Mapping: A Systematic Review of the Literature. J. Data Inf. Sci..

[45] Chen C., Leydesdorff L. (2014). Patterns of Connections and Movements in Dual-Map Overlays: A New Method of Publication Portfolio Analysis. J. Assoc. Inf. Sci. Technol..

[46] Islam M.N., Das S.R., Emin M.T., Wei M., Sun L., Westphalen K., Rowlands D.J., Quadri S.K., Bhattacharya S., Bhattacharya J. (2012). Mitochondrial Transfer from Bone-Marrow-Derived Stromal Cells to Pulmonary Alveoli Protects against Acute Lung Injury. Nat. Med..

[47] Jackson M.V., Morrison T.J., Doherty D.F., McAuley D.F., Matthay M.A., Kissenpfennig A., O’Kane C.M., Krasnodembskaya A.D. (2016). Mitochondrial Transfer Via Tunneling Nanotubes Is an Important Mechanism by Which Mesenchymal Stem Cells Enhance Macrophage Phagocytosis in the in Vitro and in Vivo Models of Ards. Stem Cells.

[48] Morrison T.J., Jackson M.V., Cunningham E.K., Kissenpfennig A., McAuley D.F., O’Kane C.M., Krasnodembskaya A.D. (2017). Mesenchymal Stromal Cells Modulate Macrophages in Clinically Relevant Lung Injury Models by Extracellular Vesicle Mitochondrial Transfer. Am. J. Respir. Crit. Care Med..

[49] Faust H.E., Reilly J.P., Anderson B.J., Ittner C.A., Forker C.M., Zhang P., Weaver B.A., Holena D.N., Lanken P.N., Christie J.D. (2020). Plasma Mitochondrial DNA Levels Are Associated with Ards in Trauma and Sepsis Patients. Chest.

[50] Athale J., Ulrich A., MacGarvey N.C., Bartz R.R., Welty-Wolf K.E., Suliman H.B., Piantadosi C.A. (2012). Nrf2 Promotes Alveolar Mitochondrial Biogenesis and Resolution of Lung Injury in Staphylococcus Aureus Pneumonia in Mice. Free Radic. Biol. Med..

[51] Gonzalez A.S., Elguero M.E., Finocchietto P., Holod S., Romorini L., Miriuka S.G., Peralta J.G., Poderoso J.J., Carreras M.C. (2014). Abnormal Mitochondrial Fusion-Fission Balance Contributes to the Progression of Experimental Sepsis. Free Radic. Res..

[52] Yuan Z., Syed M.A., Panchal D., Joo M., Colonna M., Brantly M., Sadikot R.T. (2014). Triggering Receptor Expressed on Myeloid Cells 1 (Trem-1)-Mediated Bcl-2 Induction Prolongs Macrophage Survival. J. Biol. Chem..

[53] Chang A., Ulrich A., Suliman H., Piantadosi C. (2015). Redox Regulation of Mitophagy in the Lung During Murine Staphylococcus Aureus Sepsis. Free Radic. Biol. Med..

[54] Zhang Y., Sauler M., Shinn A.S., Gong H., Haslip M., Shan P., Mannam P., Lee P.J. (2014). Endothelial Pink1 Mediates the Protective Effects of Nlrp3 Deficiency During Lethal Oxidant Injury. J. Immunol..

[55] Nakahira K., Kyung S.-Y., Rogers A.J., Gazourian L., Youn S., Massaro A.F., Quintana C., Osorio J.C., Wang Z., Zhao Y. (2013). Circulating Mitochondrial DNA in Patients in the Icu as a Marker of Mortality: Derivation and Validation. PLoS Med..

[56] Lee Y.-L., King M.B., Gonzalez R.P., Brevard S.B., Frotan M.A., Gillespie M.N., Simmons J.D. (2014). Blood Transfusion Products Contain Mitochondrial DNA Damage-Associated Molecular Patterns: A Potential Effector of Transfusion-Related Acute Lung Injury. J. Surg. Res..

[57] Boudreau L.H., Duchez A.-C., Cloutier N., Soulet D., Martin N., Bollinger J., Paré A., Rousseau M., Naika G.S., Lévesque T. (2014). Platelets Release Mitochondria Serving as Substrate for Bactericidal Group Iia-Secreted Phospholipase A2 to Promote Inflammation. Blood.

[58] Yu J., Shi J., Wang D., Dong S., Zhang Y., Wang M., Gong L., Fu Q., Liu D. (2016). Heme Oxygenase-1/Carbon Monoxide-Regulated Mitochondrial Dynamic Equilibrium Contributes to the Attenuation of Endotoxin-Induced Acute Lung Injury in Rats and in Lipopolysaccharide-Activated Macrophages. Anesthesiology.

[59] Laffey J., Matthay M. (2017). Years of Research in ARDS.Cell-based Therapy for Acute Respiratory Distress Syndrome. Biology and Potential Therapeutic Value. Am. J. Respir. Crit. Care Med..

[60] Puri G., Naura A.S. (2020). Critical Role of Mitochondrial Oxidative Stress in Acid Aspiration Induced Ali in Mice. Toxicol. Mech. Methods.

[61] Liu X., Chen Z. (2017). The Pathophysiological Role of Mitochondrial Oxidative Stress in Lung Diseases. J. Transl. Med..

[62] Singh K.K., Chaubey G., Chen J.Y., Suravajhala P. (2020). Decoding Sars-Cov-2 Hijacking of Host Mitochondria in Covid-19 Pathogenesis. Am. J. Physiol. Cell Physiol..

[63] Saleh J., Peyssonnaux C., Singh K.K., Edeas M. (2020). Mitochondria and Microbiota Dysfunction in COVID-19 Pathogenesis. Mitochondrion.

[64] Shi C.-S., Qi H.-Y., Boularan C., Huang N.-N., Abu-Asab M., Shelhamer J.H., Kehrl J.H. (2014). Sars-Coronavirus Open Reading Frame-9b Suppresses Innate Immunity by Targeting Mitochondria and the Mavs/Traf3/Traf6 Signalosome. J. Immunol..

[65] Malavolta M., Giacconi R., Brunetti D., Provinciali M., Maggi F. (2020). Exploring the Relevance of Senotherapeutics for the Current Sars-Cov-2 Emergency and Similar Future Global Health Threats. Cells.

